# Microwave-Assisted Extraction of Carrageenan from *Sarcopeltis skottsbergii*

**DOI:** 10.3390/md21020083

**Published:** 2023-01-25

**Authors:** Milena Álvarez-Viñas, Sandra Rivas, María Dolores Torres, Herminia Domínguez

**Affiliations:** CINBIO (Centro de Investigaciones Biomédicas), Facultade de Ciencias, Universidade de Vigo, Campus Ourense, Edificio Politécnico, As Lagoas, 32004 Ourense, Spain

**Keywords:** red seaweed, carrageenan, chemical-free extraction and precipitation, molecular weight, rheology

## Abstract

The development of greener processes for the sustainable utilization of raw materials is increasingly demanded for environmental and economic reasons. A rapid and chemical-free technique was proposed for the extraction of hybrid kappa/iota (6/4) carrageenan from *Sarcopeltis* (*ex Gigartina*) *skottsbergii*. After separation, carrageenans were analyzed by Fourier transform infrared attenuated total reflectance, high-performance size-exclusion chromatography, and rheology. Maximum carrageenan extraction yields up to 63–64% were obtained operating at 110 or at 160 °C, for 5–7 min considering the sum of the heating and cooling periods, but the extraction of the phenolic fraction was favored at 220 °C. The recovered carrageenan showed apparent viscous values around 10^3^ mPa at the lowest tested shear rates (0.1 1/s) and could be suitable to formulate films. Furthermore, those carrageenans obtained under 140 °C showed gel characteristics without previous separation from the liquid extract, avoiding ethanolic precipitation and energy consumption. The antiradical properties correlated with the phenolic content in the liquid phase, but no influence of temperature on the reducing properties was observed. The microwave-assisted hydrothermal treatment could be an efficient tool without needing chemicals for the extraction of carrageenans, which showed adequate rheological properties for commercial uses.

## 1. Introduction

Carrageenan is a linear polysaccharide with a disaccharide of D-galactose and 3,6-anhydro-galactose (3,6-AnGal) as the basic unit, and with sulfate groups in number and position differing among the different carrageenan families, being kappa, iota, and lambda, the most commercially relevant [1]. In red seaweeds, carrageenans are found as mixtures of different types. They are used as thickening, gelling, and texturing ingredients in food, cosmetics, and in pharmaceuticals to prepare microstructures for controlled drug administration [2].

The high molecular weight polymers (usually 200–800 kDa) are approved for food uses [3]. However, low molecular weight carrageenans (10–40 kDa) are not authorized for these purposes since they cause different inflammatory responses [4,5], and show many interesting activities, including antiviral, antitumoral, antibacterial, and immunostimulant [6,7,8]. These biological properties of carrageenans are dependent on their type and structure, sulfation degree and location, polymerization degree, and processing methods [9].

Carrageenans are generally extracted with hot aqueous solutions using water or mild alkaline media. Different intensification technologies have been applied for the reduction in chemicals, including pressurized hot water extraction [10,11] and microwave heating pretreatment before [12], or during, pressurized hot water extraction [13]. This technology could be incorporated after extraction of the lipidic and phenolic fractions with supercritical CO_2_ [14]. These strategies were applied to *Mastocarpus stellatus* and allowed reduced operation time and energy consumption compared to the conventional extraction or lower alkali concentration during pretreatment [12].

In a previous study on *Chondrus crispus*, Álvarez-Viñas et al. (2022) [11] tried a simultaneous extraction and depolymerization of carrageenan during hydrothermal treatment at temperatures in the range 120–200 °C and the microwave assistance succeeded for the further recovery of *C. crispus* carrageenan from the industrial wastes after extraction of hybrid carrageenan [15].

Carrageenan oligosaccharides show enhanced bioavailability and bioactivity and can be produced from the polymer using hydrolysis by chemical (mild acid hydrolysis, free radical depolymerization), enzymatic, or physical [1,16,17] means. Chemical hydrolysis is simple, low-cost, rapid, and scalable, but has disadvantages derived from its low specificity, the production of side-products, the associated environmental hazards [18], and it can also yield fragments with very low molecular weight (1.2–3.0 kDa) and decrease the sulfate and 3.6-AnGal contents [16]. Enzymatic depolymerization is environmentally friendly and advantageous, since the resulting oligosaccharides show higher homogeneity and lower polydispersity than with chemical methods [18,19], and it produces low amounts of monosaccharides and toxic molecules. However, it requires a long reaction time and the cost of enzymes is high [18]. Autohydrolysis has also been applied to conventionally extracted carrageenans [20].

Alternative and more recent solutions have been suggested, including microwave heating to enhance the acid depolymerization of native κ-carrageenan and to shorten the reaction time. Bouanati et al. (2020) [21] proposed a 5 min controlled microwave-assisted hydrolysis in pure water for the production of bioactive oligosaccharides from both commercial carrageenans and from carrageenans from *Kappaphycus alvarezii* or *Eucheuma spinosum*, preserving the sulfate groups. However, severe operation conditions can cause excessive loss of sulfate groups, which are key for some biological properties [21].

Microwave-assisted pressurized hot water extraction has been useful with seaweeds containing predominant structures of kappa carrageenan and hybrid mixtures of kappa/iota [11,12,13,15]. Other seaweeds contain a wide variety of carrageenan types; *Sarcopeltis* (*ex Gigartina*) *skottsbergii* has been reported to contain mainly κ-carrageenan, with significant amounts of ι- and ν-carrageenan [22], or a mixture α-, β-, ι-, κ-, and ξ- [23,24].

The present study aims at evaluating the possibility of using a microwave-assisted extraction of carrageenan from *S. skottsbergii* and the effects on the process performance and properties of the products.

## 2. Results

### 2.1. Characterization

The proximal composition of *Sarcopeltis skotsbergii* specimens used in the present study is summarized in Table 1. The extractives, ash, and protein content account for 6.90, 16, and 10% of the dry weight, respectively. Carbohydrates, expressed as polymers, represent 52.08% of the total content, galactose being the main saccharide constituent, which accounts for more than 40%. Regarding mineral content, sodium (23.2 g/kg) is by far the most abundant, followed by potassium (11.7 g/kg), magnesium, (7.2 g/kg), and phosphorus (1.3 g/kg). Iodine, zinc, iron, and calcium are found in minor amounts.

### 2.2. Influence of Extraction Temperature

#### 2.2.1. Crude Extract

**Figure 1:**
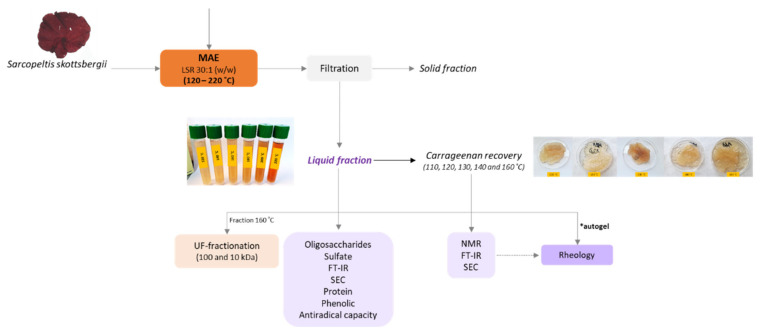
Flow diagram of the process proposed for the microwave-assisted water extraction of carrageenan and analytical stages. * The liquid fractions of temperatures lower than 140 °C presented hydrogel-like rheological properties, named autogel.

Figure 1 shows a flow diagram with the sequence of stages for processing *S. skottsbergii* by microwave-assisted water extraction and the analytical characterization of the resulting streams. The dry commercial seaweed was subjected to extraction with distilled water. The solid and liquid phases were separated by vacuum filtration and the carrageenan in the liquid phase was further separated by ethanol precipitation. The carrageenan was chemically and structurally characterized and then was used for the formulation of films. Precipitation was not observed from the extracts obtained at temperatures higher than 160 °C. However, the soluble fraction obtained at temperatures under 140 °C showed hydrogel-like rheological properties, without needing the ethanolic precipitation and further dissolving stages.

**Figure 2:**
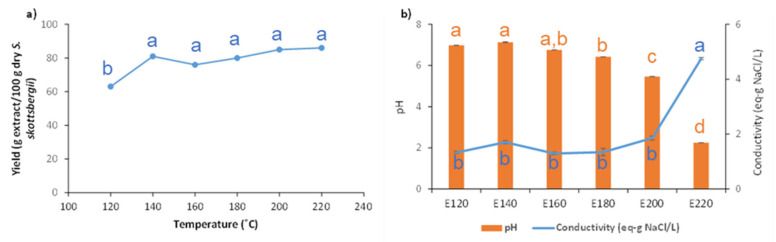
Influence of the treatment temperature on (**a**) total extraction yield and (**b**) conductivity and pH during microwave-assisted extraction of *S. skotsbergii.* Data values for each series with different superscript letters are statistically different (*p* ≤ 0.05).

A marked increase in the overall extraction yield was observed up to 140 °C, and then the values increased slightly up to 85% at 220 °C (Figure 2). The conductivity was relatively constant with temperature and was maintained under 2 g NaCl eq/L in the range 120–200 °C, and increased abruptly at 220 °C to a value of 4.5 g NaCl eq/L. The pH of the liquid extract obtained after the hydrothermal treatment was neutral at 120–140 °C and slightly acidic in the range 160–180 °C, but was progressively acidified at 200 °C and more markedly, with pH 2, at 220 °C.

**Figure 3:**
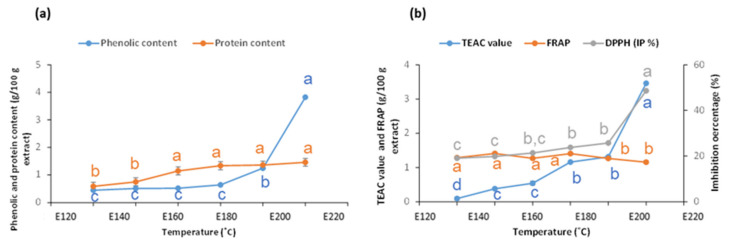
Influence of the hydrothermal treatment temperature on (**a**) phenolic (expressed as gallic acid equivalents) and protein content in the soluble fraction and (**b**) antioxidant properties in the soluble fraction. Data values for each series with different superscript letters are statistically different (*p* ≤ 0.05).

The influence of extraction temperature on the phenolic content (expressed as gallic acid equivalents per 100 g extract) of the extract (Figure 3a) showed a continuous increase from 0.5 g/100 g to more than 1 g/100 g at 200 °C, and a more marked increase up to 4 g/100 g operating at 220 °C. Data confirm a very slight increase with operation temperature up to 180 °C in the protein content of the extracts, which then moderated and remained under 2% of the dry weight. Whereas the reducing power of ferric ion remained almost unaffected by the extraction temperature, the antiradical properties increased markedly at the highest temperatures (Figure 3b), following an expected trend with the phenolic content.

**Figure 4:**
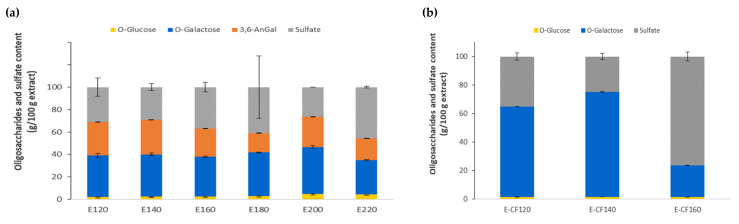
Influence of the hydrothermal treatment temperature on saccharidic constituents, and sulfate and reaction products content in (**a**) the crude extract and (**b**) in the carrageenan-free extract obtained at 120–160 °C.

Data in Figure 4a show the relative content of each saccharidic constituent and sulfate in the dried crude extract confirming that both the galactose and sulfate proportions increased with the treatment temperature. However, the maximum absolute values of galactose (54.00 ± 0.50 %), glucose (3.92 ± 0.41 %), and sulfate (56.89 ± 27.96 %) were found in the product obtained at 180 °C. The error associated with the sulfate determination in the crude extracts was too high in all experiments, but the sulfate content significantly decreased at temperatures higher than 180 °C. In the carrageenan-free liquid phase, a corresponding increase with increasing temperature was noticed at 160 °C (Figure 4b). According to the SEC profiles (Figure S1), the crude extracts showed peaks higher than 800 kDa and lower than 14 kDa. The degradation at higher temperatures could be also visualized after membrane fractionation in 100 kDa and 10 kDa membranes of the extracts obtained at 160 °C. Only 6% of the material showed a molecular weight higher than 100 kDa, 81% of the extract was retained between 10 and 100 kDa, and 13% was under 10 kDa.

**Figure 5:**
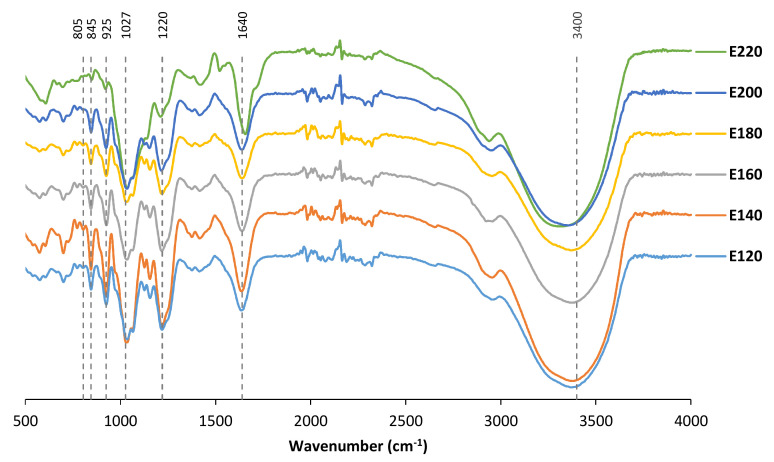
Influence of hydrothermal treatment on *S. skottsbergii* crude extracts on FT-IR profiles.

**Figure 6:**
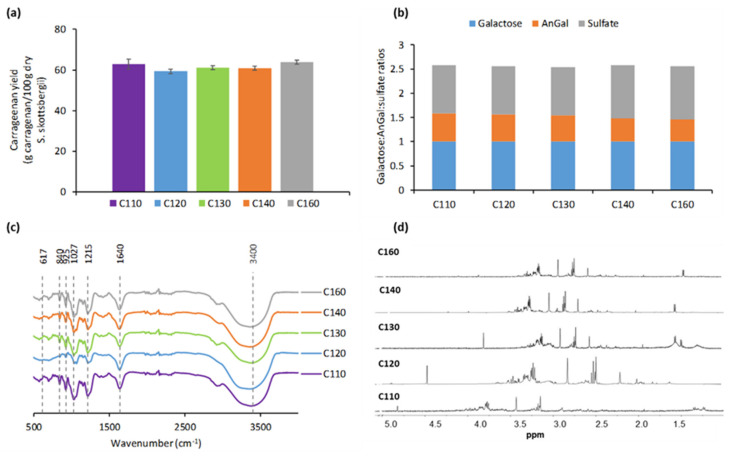
Characterization of the extracted carrageenan (**a**) extraction yield; (**b**) Gal:AnGal:sulf ratio; (**c**) FT-IR profiles; and (**d**) H-NMR spectra.

Fourier Transform Infrared Attenuated Total Reflectance profiles exhibited a typical kappa band of about 845 cm^−1^ attributed to D-galactose-4-sulfate (Figure 5 and Figure 6c). A characteristic iota band around 805 cm^−1^ ascribed to the presence of a sulfate group located on the second carbon of the anhydro ring was also identified. A reasonably strong peak of about 925 cm^−1^ confirmed the existence of 3,6-anhydro-D-galactose. Extracted hybrid carrageenans also featured a relevant peak of about 1215 cm^−1^ related to the S=O stretching vibration of the sulfated groups [25]. The observed peak at 1500 cm^−1^ is attributed to the sulfate stretching [26].

#### 2.2.2. Crude Carrageenan

The crude carrageenan in extracts from 120 and 140 °C showed hydrogel characteristics without requiring precipitation and dissolving stages. This interesting finding led to the expansion of the range of extraction temperatures for the analysis of carrageenan to 110 and 130. This fact could be ascribed to the presence of salts in the liquid medium (Table 2), especially the ions K, Ca, Mg, and Na.

Maximum recovery yields of non-purified carrageenans were obtained from crude extracts produced at 110 °C, with 62.87 ± 2.49%, 59.28 ± 2.43% at 120 °C, 61.11 ± 1.57% at 130 °C, 60.84 ± 2.2% at 140 °C, and 63.79 ± 0.27% at 160 °C (Figure 6a). Data in Figure 6b confirm that the galactose:anhydrogalactose:sulfate ratio was maintained, with values of 1.0:0.58:1.01 for C110; 1.0:0.56:1.02 for C120; 1.0:0.54:1.04 for C130; 1.0:0.48:1.11 for C140; and 1.0:0.46:1.18 for C160. Data in Figure 6c show typical functional groups of kappa/ iota-hybrid carrageenan, also confirmed by the ^1^H-NMR profiles in Figure 6d.

Independently of microwave thermal conditions, the kappa- and iota-carrageenan molar fractions determined by ^1^H NMR accounted for around 60 ± 3 % and 40 ± 3 %, respectively. The spectra of tested carrageenans showed peaks around 1.44 ppm indicating the presence of impurities attributed to methyl protons of the cyclic pyruvate; in addition, minor pigments were identified in the samples [27].

The intensity of the GPC peaks showed a progressive decrease with extraction temperature, both in the peaks corresponding to more than 800 kDa and in those lower than 24 kDa (Figure S2). This behavior suggests higher resistance to flow for those carrageenans extracted under the mildest processing conditions. The mild acidic conditions generated in the media during the hydrothermal process could lead to the fragmentation of glycosidic linkages. A similar trend was reported for the autohydrolysis of a partially cyclized mu/nu-carrageenan from *S. skottsbergii* caused after 11 h at 60 °C, from the initial 198 kDa carrageenan to fractions lower than 1.9 kDa [28].

**Figure 7:**
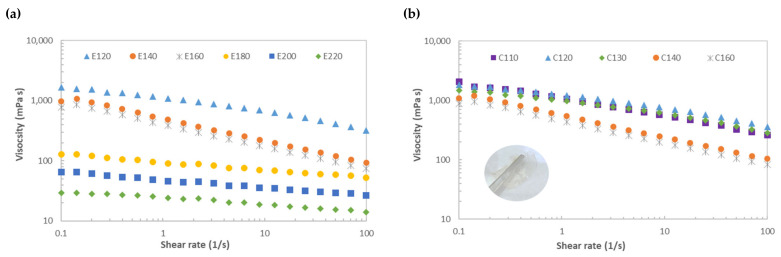
Viscous profiles of the *Sarcopeltis skottsbergii* (**a**) crude extracts and (**b**) extracted hybrid carrageenan. Inset in plot b is a representative film prepared with C120 given its higher extraction yield.

Figure 7a shows the impact of the microwave-assisted water extraction on the viscous profiles of the crude extracts obtained from *Sarcopeltis skottsbergii.* The apparent viscosity of the crude extracts decreased with increasing shear rate over the tested range. This shear thinning behavior was favored for crude extracts recovered at the lowest microwave thermal conditions. It should be noted that viscous profiles of crude extracts obtained above 180 °C tended to Newtonian behavior. At the fixed shear rate, the strongest viscous behavior was observed for E120, followed by E140 and E160 > E180 > E200 > E220.

Data in Figure 7b display the viscous profiles of the corresponding extracted hybrid carrageenans. All extracted biopolymers exhibited shear thinning behavior, with relatively higher apparent viscosity values (about double) than those identified for the crude extracts. Shear thinning was promoted for the biopolymers treated at the highest microwave temperatures, i.e., C140 and C160. This suggests that lower average molecular weights can be found in these systems (Figure S2) since the flow can be favored at the highest shear rates due to a better alignment of the smaller size fractions present in the biopolymer. At the fixed shear rate, C110 featured the highest apparent viscosity without notable differences with C120 and C130, followed by C140 and C160. It should be highlighted that no thixotropic phenomena were identified in any of the tested samples.

#### 2.2.3. Residual Solids

**Figure 8:**
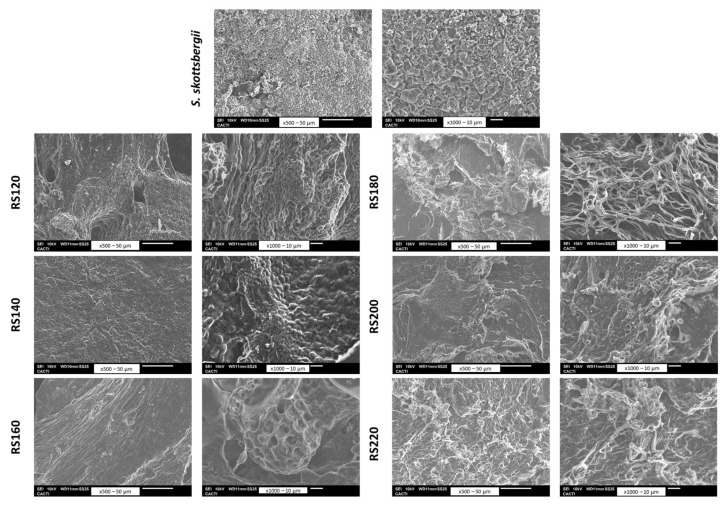
SEM photos of the untreated seaweed and the residual solids after microwave-assisted hydrothermal treatment at tested temperatures: RS120–RS220.

Data in Table 3 summarize the protein content of the residual solids, confirming and enriching by three times compared to the initial seaweed. Figure 8 shows the microstructure of the initial seaweed and the residual solids, confirming that a more porous structure was observed after treatments at 160 and 180 °C.

## 3. Discussion

The carbohydrate fraction is the most abundant but the protein content in S. *skottsbergii* accounted for around 10%, in the range (5.5–7.6%) reported for this species [29,30]; although, higher values are usually reported for other red seaweeds. This macroalga exhibits a remarkable content of the highly valuable R-phycoerythrin protein [30]. Total carbon content was higher than nitrogen and the C:N index was 18.4, slightly lower than 23.8, reported for these seaweeds [30]. Low lipid content, 0.2%, has been reported for this species [29]. The ash levels in the seaweed samples used are 16%, lower than the 26% reported for specimens from Chilean central and southern regions and industrially dried for commercial purposes [29]. The high iron levels are closely dependent on the collection area and have been related to changes in the oxidative cellular balance [31]. Arsenic levels are lower than the range found in many commercial products made from commercial seaweeds [32] and also lower than the values reported for this species collected from Chilean coastal zones [33].

As expected, the extraction yields were higher than the 30% attained with water at room temperature [22], 50% with water at 90 °C during 3 h in two stages [34], 50–55% by ultrasound-assisted treatment at 150 W and 90 °C during 15 min [35], or the 68% with water extraction at 100 °C for 24 h and the 42% during an ultrasonic-assisted water extraction (75 W, 20 kHz, 60 min) [24]. Short treatment times have been reported for microwave-assisted extraction and hydrolysis treatments to produce bioactive oligosaccharides. Bouanati et al. (2020) [21] proposed a 5 min microwave-assisted hydrolysis from both commercial carrageenans and from carrageenans from *Kappaphycus alvarezii* or *Eucheuma spinosum*, preserving the sulfate groups to produce sulfated oligosaccharides with DP under 25. Ponthier et al. (2020) [13] reported good viscoelastic behavior of hydrogels formulated with carrageenan extracted by microwave assistance at 170 °C for 6 min.

The increased conductivity obtained when the autohydrolysis temperature was increased from 120 to 140 °C probably corresponded with some alkaline elements, whereas the more pronounced increase in extracts obtained at temperatures higher than 200 °C was probably due to the sulfate groups released, which also caused acidification in the medium and to the lowered sulfate content in the carrageenan phase (Figure 4a) and the high sulfate content in the carrageenan-free extract (Figure 4b). The amount of sodium, potassium, calcium, and magnesium ions present in the soluble extracts corroborated the observed conductivity trends. The presence of these ions in the liquid phases recovered after hydrothermal treatment below 160 °C seems to generate sufficient ionic strength in the medium for the spontaneous gelling of the hybrid carrageenan. This self-gelling achievement involves relevant advantages from the industrial point of view. It allows for saving resources in the absence of the conventional ethanolic precipitation stage (ethanol: hydrothermal liquid phase, 2.5:1 *v/v*) [36]. It can also involve a reduction in energy consumption (estimated at about 4 × 10^3^ kJ) by avoiding the biopolymer drying stage around 24 h [37], without jeopardizing the rheological features of the obtained gels at comparable ionic strengths [38].

Although it was not the objective, the protein extraction yield was around 1%. Castro-Varela et al. (2022) [30], in a process for the valorization of the R-phycoerythrin fraction, after cell disruption with ultrasound-assisted extraction and high-pressure homogenization (100–400 MPa in 2–3 passes), obtained up to 5.5 mg/g, which could be further concentrated through ultrafiltration in 30 kDa membranes. The lowered N content in the extracts was also reported with other technologies, i.e., in water extracts assisted by ultrasound at 100 °C for 24 h [24].

The carrageenan recovery yield in the proposed process was high. In a previous study with conventional heating, up to 90% of the initial mass was solubilized operating under a non-isothermal regime during heating up to 200 °C, and a maximum crude carrageenan yield of 75% was attained at 140 °C [11]. In that study, crude carrageenan could not be precipitated by ethanol from the extracts produced at more than 180 °C, but ultrafiltration performed well regarding carrageenan recovery yield and properties. From *S. skottsbergii*, other authors have reported the use of isopropyl alcohol, i.e., Matulewicz et al. (1989) [39] after extraction at room temperature for 16–24 h and isopropanol precipitation obtained yields in the range 51–53%, and for Westermeier et al. (2002) [23], after washing with 3% potassium chloride solution and then extraction with hot water at 90 °C for 2 h, precipitation with 97% isopropyl alcohol yielded 38–59% depending on location and season.

The structure of extracted biopolymers is consistent with that previously found for the carrageenans recovered from *S. skottsbergii* red seaweed treated under conventional procedures [40] and other hybrid carrageenans extracted from different sources using alkaline conditions [25,41]. These outcomes confirmed that *S. skottsbergii* red seaweed is fundamentally a carrageenophyte seaweed from which it is possible to recover kappa/iota-hybrid carrageenan, even though the biopolymer isolation is conducted in the absence of alkali pre-treatment. Samples from 220 °C showed differences in the bands between 1000 and 1200 cm^−1^, as already reported by Saluri and Tuvikene (2020) [20], for degradation during autohydrolysis. The signal around 1200 cm^−1^ was lowered at 220 °C, suggesting the loss of sulfated units [20].

Values of the molar fractions of kappa- and iota-carrageenan disaccharide units determined by ^1^H NMR agreed with those reported for other carrageenophyte seaweeds such as *S. crispata* (kappa: 58, iota:42) [42], *M. stellatus* (kappa: 69, iota: 31) [36], or *C. crispus* (kappa: 78, iota: 22) [43], where alkali processing conditions were employed. The thermal treatment used during the microwave-assisted extraction seems to impact the purity of the recovered carrageenans since the impurities found in the samples tended to increase with increasing thermal processing conditions during the biopolymer extraction [44].

The composition (galactose, anhydrogalactose, and sulfate groups) and structure (molecular weight) strongly influence the technological and biological properties of these polysaccharides. Barahona et al. (2012) [34] reported that the water extraction (90 °C, 3 h, two stages) from the green variant of tetrasporic *Sarcopeltis skottsbergii* yielded 50% polysaccharide, and the retentate after dialysis at 3.5 kDa and ethanol precipitation led to a product containing 64% reducing sugars, 26% of sulfate, and 2.5% of protein. Extraction with water at room temperature provided a product with 58% carbohydrates, 30% sulfate, and 4% protein, with a Gal:AnGal:sulfate ratio of aqueous extracts 1.0:0.6:1.2 [22], which is consistent with the ratio found here. These ratios also vary among the cystocarpic and tetrasporangial plants of *Sarcopeltis skottsbergii* [39]. During the thermal decomposition of carrageenan in a sol state, the sulfation degree and the 3,6-anhydrogalactose content influenced the amount of 5-hydroxymethylfurfural (yield 0.7–21.8%) [44]. In our study, no degradation products were detected in the direct chromatographic analysis.

The phenolic yield from the dry seaweeds accounted for 0.4 to 3.2 g/100 g, confirming that the selected treatments were more effective than conventional solvent extraction. Guinea et al. (2012) [45] extracted chlorophylls and carotenoids with acetone twice for 2 h and the extracted phenolics by 70% acetone at room temperature for 1 h and at 6 °C overnight and obtained 0.29 g/kg seaweed. They confirmed that these compounds were safe for embryos and showed photoprotective action, which could also be due to other components, such as mycosporine-like amino acids. Ortiz-Viedma et al. (2021) [29] extracted 1.13 g phenolics/100 g seaweed when they used 50% ethanol with shaking for 8 h and then sonication in a bath at 25 °C during 15 min and stirred for an additional 15 min in a process repeated in three stages. They found that *S. skottsbergii* ethanolic extract showed the highest antiradical activity (DPPH, FRAP) among other Rhodophyta and was effective against the lipid oxidation of salmon paste, in the formation of primary oxidation products, and in the protection of the endogenous antioxidants, tocopherols and astaxanthin, as well as showing antimicrobial action. Other compounds could also contribute to the antioxidant activity; i.e., Castro-Varela et al. (2022) [30] confirmed that R-phycoerythrin showed a positive correlation with antioxidant capacity.

The antioxidant properties of the extracts from the present study have been analyzed as the DPPH and ABTS radical scavenging capacity and the reducing power, assessed as the ferric reducing antioxidant capacity. Whereas the antiradical properties increased with temperature with the phenolic content in the extract, the reducing properties remained almost constant, regardless of the extraction temperature. This property was also reported in other extracts [29]. The IC_50, DPPH_ for the extract obtained at 220 °C was 24.68 g/L, a value lower than for pure phenolic synthetic products, IC_50,BHA_= 0.24 g/L and IC_50,BHT_ = 2.4 g/L, but in the range of those of extracts from other red seaweeds [46]. With *Mastocarpus stellatus* having the highest phenolic yield, antioxidant capacity and sulfate content were also observed at 190 °C in operation with microwave-assisted extraction in a short heating time (3 or 6 min) [13].

Barahona et al. (2012) [34] isolated a water-soluble polysaccharide from the green variant of tetrasporic *S. skottsbergii,* which was composed of D-galactose and sulfate groups in a molar ratio of 1.0:0.65, a similar proportion to λ carrageenan but with lower sulfation. This carrageenan showed higher hydroxyl and ABTS^+^ radical scavenging capacity than commercial λ carrageenan, but lower antiradical action against peroxyl radicals due to the lower sulfate content.

The method proposed in the present study operates in very short times, around 10 min during heating and cooling, and maintains molecular weights higher than 780 kDa. However, the presence of a fraction under 23.6 kDa would not recommend its use for food applications [4,5]. The polysaccharides extracted with water reflux for 24 h showed lower molecular weight (78 kDa) than the solubles extracted with ultrasound assistance at 75 W and 20 kHz for 60 min (565 kDa) or with carbonate/peroxide assisted by ultrasound (994 kDa) [24]. Aqueously extracted carrageenan with water at 90 °C for 3 h, showed a molecular weight of 1000 kDa [34].

Similarly, Saluri and Tuvikene (2020) [20] reported that for θ-carrageenan, the reducing properties showed an increase with a decrease in molecular weight, due probably to other changes in the polysaccharide chain. However, scarce effects were observed on the λ-carrageenan. Optimal values could be found at 15 kDa for θ-carrageenan and 280 kDa for λ-carrageenan samples; the latter being less active, and in both cases, no significant improvement could be found with lower molecular sizes. Other features could influence the antioxidant activity of carrageenans, such as the sulfate content or the anhydrogalactose content, which enhances antioxidant properties in lower molecular weight samples. They reported the antioxidant and anticoagulant activities of both native λ-carrageenan and θ-carrageenan obtained after alkali treatment and their products of autohydrolysis, which is a mild treatment, but it could be as long as 72 h. In this treatment, the molecular weight of λ-carrageenan was lowered from 3100 to 4.7 kDa, and that of θ-carrageenan from 630 to 1.1 kDa.

Variations in viscosity have been observed with location, seasonality, and life stages from gametophytes up to 120 mPa, and from tetrasporophytes up to 1000–1400 mPa [23]. The magnitude of the apparent viscosity of hybrid carrageenan found in this work is between 1000 and 2000 mPa, which is consistent with those values found suitable for the development of biofilms from other natural biopolymers such as mixtures of carrageenan and chestnut starch [47] or potato starch [17]. These viscosity values drop around a factor of ten for crude extracts under 180 °C of processing conditions. The absence of hysteresis in the extracted hybrid carrageenans is another advantage from the industrial manufacturing point of view.

## 4. Materials and Methods

### 4.1. Materials

Dehydrated *Sarcopeltis skottsbergii* red seaweed was kindly provided by CEAMSA Company (Porriño, Pontevedra, Spain). The seaweed was stored in sealed plastic bags in a dark environment until further use.

### 4.2. Microwave-Assisted Water Extraction

Microwave-assisted water extraction was carried out in a Monowave 450 model microwave reactor (Anton Parr GmbH, Graz, Austria). Fast heating was used for all tests, accounting for 1–1.6 min, and cooling time varied between 5 and 6 min. A solid:liquid ratio of 1:30 (*w/w*) was maintained, and the maximum temperatures assayed were between 110 and 220 °C. The mixture was stirred at 900 rpm in a standard 30 mL Pyrex container, and temperature was determined through an IR detector.

### 4.3. Physicochemical Characterization of the Raw Material

Raw material composition was determined following the standardized methods: extractives [48] (NREL/TP-510-42619), ash [49] (NREL/TP-510-42622), structural carbohydrates, acetyl groups, and acid insoluble residue (AIR) [50] (NREL/TP-510-42618). In the methodology NREL/TP-510-42618 [50], two quantitative acid hydrolysis are involved, which provide a soluble liquid phase and a solid (insoluble) one. Then, the liquid fraction was filtered through 0.45 µm membranes and analyzed by High-Performance Liquid Chromatography (HPLC) (1100 series, Agilent, Santa Clara, CA, USA) using an Aminex HPX-87H column (300 × 7.8 mm, BioRad, Hercules, CA, USA) operating at 60 °C with 0.003 M H_2_SO_4_ at 0.6 mL/min as mobile phase, whereas the solid fraction remaining was quantified as AIR.

The sulfate content was studied using the ion chromatography technique (Metrohm Advanced IC-861, Herisau, Switzerland). In order to assess the mineral and heavy metal content, an acid digestion (HNO_3_ and H_2_O_2_ at 1600 W for 15 min and 200 °C for 10 min) was conducted on a Marsxpress microwave (CEM, Charlotte, NC, USA). Na and K contents were determined by Atomic Emission Spectrophotometry (AES); Zn, Ca, Mg, Fe, and Cu were evaluated by Atomic Absorption Spectrophotometry using a 220 Fast Sequence Spectrophotometer (Varian, Palo Alto, CA, USA), and Cd and Pb by using Inductively Coupled Plasma MS (ICP-MS) (X Series, Thermo Scientific, Waltham, MA, USA). Total nitrogen content was assessed by the Kjeldahl method and the value was converted to protein using the factor 4.92 (average value specific for red algae) [51].

Scanning electron microscopy measurements were performed in both the seaweed used as raw material and the corresponding solid phases obtained after microwave-assisted water extraction. Seaweeds were placed on aluminum beads before being plated using an Emitech K550X (Quorum Technologies Ltd., Lewes, UK). All samples were monitored with a QUANTA 200 FEI (Field Emission Ion) Scanning Electron Microscope (SEM) (FEI Company, Eindhoven, The Netherlands) using vacuum processing conditions with accelerating voltage (15.0 kV). Measurements were run at least in duplicate.

### 4.4. Chemical Characterization of the Liquid Phase and Carrageenan

The determination and quantification of oligosaccharides were carried out for all the recovered liquid fractions and the corresponding carrageenan by means of a post-hydrolysis with sulfuric acid at 4% *w/w* and 121 °C for 20 min in an autoclave. It was analyzed by HPLC as aforementioned. Note, here, that the carrageenan was previously precipitated from the recovered liquid phases using 96% ethanol in a ratio of 1:1.5 (liquid phase:ethanol).

### 4.5. Total Phenolic Content, Sulfate Content, Protein Content, and Antioxidant Capacity Assays

The total phenolic content was determined following the Folin–Ciocalteu method [52]. The Folin–Ciocalteu reagent (Scharlau, Spain) and 20% sodium carbonate were used, and incubated for 1 h in the dark and at room temperature. It was measured at 730 nm using UV–Visible Spectrophotometer Evolution 201 (Thermo Scientific, Waltham, MA, USA). The standard curve was prepared with gallic acid (Sigma, Saint Louis, CA, USA).

The soluble sulfate content was determined by the Dodgson gelatin–barium chloride method [53]. Trichloroacetic acid (TCA, 4%) (Sigma-Aldrich, Spain) and a gelatin–barium chloride mixture were used as reagents. The absorbance was measured at 500 nm.

The soluble protein was quantified with the Bradford reagent. The samples were incubated for 15 min at room temperature. Absorbance was measured at 595 nm and compared to a standard curve prepared with bovine serum albumin (BSA) (Sigma, Saint Louis, CA, USA).

The antioxidant capacity was determined by the Ferric Reducing Antioxidant Power (FRAP) method described by Benzie and Strain (1996) [54] as the reduction of Fe^3+^ to Fe^2+^. The FRAP reagent was prepared with 300 mM acetate buffer and a solution of 10 mM TPTZ in 40 mM HCl and 20 mM FeCl_3_·6 H_2_O in distilled water. This analysis was performed at room temperature, measuring the absorbance at 593 nm after 6 min of incubation. Ascorbic acid was used as standard reagent.

The radical cation ABTS [2,2-azinobis(3-ethyl-benzothiazoline-6-sulfonate)] (ABTS^⋅+^) was determined by the method of Re et al. (1999) [55]. The absorbance was measured at 734 nm after incubation at 30 °C of samples in the ABTS^⋅+^ solution diluted with PBS buffer for 6 min and the values were expressed as TEAC value (Trolox equivalent antioxidant capacity).

The scavenging of 1,1-diphenyl-2-picrylhydrazyl (DPPH) radicals was evaluated according to von Gadow et al. (1997) [56]. A methanolic solution (6 × 10^−5^ M) of DPPH was used in a methanolic solution of the antioxidant and the decrease in the absorbance at 515 nm after 16 min was recorded. The inhibition percentage (IP) was calculated using the following equation: IP (%): ((Abs t = 0 min) − (Abs t = 16 min)/(Abs t = 0 min)) × 100.

All the above assays were performed at least in triplicate.

### 4.6. High-Performance Size Exclusion Chromatography

High-resolution size exclusion chromatography (HPSEC) was employed to assess the molar mass distribution of the liquid fractions and crude carrageenan obtained. A serial two-column arrangement (6 × 150 mm TSKGel SuperMultipore PW-H from Tosoh Bioscience, Griesheim, Germany) equipped with a TSKGel SuperMP (PW)-H (4.6 × 35 mm) protection column in a 2-column array was used with refractive index as detector. Milli-Q water was used as mobile phase at a flow rate of 0.4 mL/min and at 40 °C. Poly(ethylene oxide) with different molecular weights (2.36 × 10^4^ − 7.86 × 10^5^ g/mol) (Tosoh Bioscience, Tokyo, Japan) were used as standards. Measurements were made at least in duplicate.

### 4.7. Fourier-Transform Infrared Spectroscopy

The lyophilized liquid fractions and the extracted crude carrageenan were mixed with KBr and analyzed by Fourier Transform Infrared Attenuated Total Reflectance (FTIR-ATR) (Nicolet 6700, Thermo Scientific, Waltham, MA, USA) equipped with a DTGS KBr detector, and the software used was OMNIC (Thermo Scientific, Waltham, MA, USA). The spectra of the samples were recorded in the selected range of 500 to 1500 nm (spectral resolution: 4 cm^−1^ and 32 scans min^−1^). Samples were analyzed at least in duplicate.

### 4.8. Proton Nuclear Magnetic Resonance

Proton Nuclear Magnetic Resonance (^1^H NMR) of the extracted carrageenan was conducted at 75 °C on a Bruker ARX400 spectrometer (Bruker, Billerica, MA, USA) operating at 400 MHz. Spectra were recorded using deuterium oxide (D_2_O) as a solvent and 3-(trimethylsilyl)-1-propane sulfonic acid (TMS-PSA, Sigma Aldrich) as internal standard. Measurements were performed at least in duplicate.

### 4.9. Membrane Fractionation

The selected extract obtained from the microwave hydrothermal treatment at 160 °C was processed on a series of ultrafiltration membranes in order of decreasing cutoff of 100 and 10 kDa (Merk-Millipore, Burlington, MA, USA). It was operated in concentration mode at a final volume concentration ratio (VCR) of 7.

### 4.10. Rheology

Steady-state shear measurements of the recovered liquid phases and the corresponding carrageenans were conducted at least in duplicate on a controlled stress-rheometer (MCR 302, Paar Physica, Graz, Austria) equipped with a Peltier system (± 0.01 °C). The selected measuring geometry was a sandblasted plate–plate (25 mm diameter, 1 mm gap) in order to prevent possible slippage during shear. Samples were placed between the rheometer plates, sealed with light paraffin oil, and left to stand for 15 min at 25 °C before rheological testing. Apparent viscosity was monitored versus increasing shear rate and just back to the initial low shear rate in order to assess the possible thixotropic effects.

### 4.11. Statistical Analysis

One-factor analysis of variance (ANOVA) was employed to evaluate the significant differences among means of experimental data. In order to differentiate average values with 95% confidence (*p* < 0.05), a post hoc Scheffé test was performed. The software PASW Statistics v.22 (IBM SPSS Statistics, New York City, NY, USA) was used.

## 5. Conclusions

The microwave-assisted pressurized hot water extraction of carrageenan from *Sarkopeltis skottsbergii* is a greener and more rapid process compared to conventional alkaline extraction. Furthermore, the addition of chemicals for extraction and precipitation is not required; the final crude carrageenan product showed adequate rheological properties and, under selected extraction conditions, gelling properties were spontaneously observed. The liquid phase remaining after carrageenan separation contained phenolic compounds with antioxidant properties. The solid residue was enriched in protein and could be further valorized. Additional ongoing studies for the valorization of other fractions than carrageenan are aimed at a more rational utilization of the feedstock, and this information will also be valuable for other red seaweeds.

## Figures and Tables

**Table 1:** Characterization of the raw material *Sarcopeltis skottsbergii,* expressed in dry basis, except moisture, which is expressed in wet basis.

Component	Content	Minerals (mg/kg)	Calcium (Ca^2+^)	4.38 ^i^
Moisture (%, w.b.)	10.19 ± 0.05 ^d^		Potassium (K^+^)	11688 ^b^
Ash (%, d.b.)	16.12 ± 0.12 ^c^		Magnesium (Mg^2+^)	7172 ^c^
AIR (%, d.b.)	2.19 ± 0.10 ^h^		Sodium (Na^+^)	23186 ^a^
Protein (%, d.b.)	10.14 ± 1.11 ^d^		Phosphorous (P^3−^)	1256 ^d^
Carbon (%, d.b.)	31.84 ± 0.40 ^b^		Zinc (Zn^2+^)	45.70 ^g^
Hydrogen (%, d.b.)	5.34 ± 0.12 ^g^		Iodine (I^−^)	334 ^e^
Sulfates (%, d.b.)	9.44 ± 0.02 ^e^	**Heavy metals (mg/kg)**	Arsenic (As^2+^)	8.48 ^h^
Extractives (%, d.b.)	6.90 ± 0.20 ^f^		Cadmium (Cd^2+^)	0.31 ^k^
**Carbohydrates (%, d.b.)**			Copper (Cu^+^)	0.89 ^j^
Glucose in polymeric units	10.76 ± 0.11 ^d^		Mercury (Hg^+^)	0.03 ^ll^
Galactose in polymeric units	36.59 ± 0.04 ^a^		Iron (Fe^2+^)	58.50 ^f^
			Lead (Pb^2+^)	0.11 ^l^

Data are given as mean ± standard deviation, except for minerals and heavy metals where standard deviations were < 2% in all cases and were independently studied. Data values in a column with different superscript letters are statistically different (*p* ≤ 0.05).

**Table 2:** Metal composition of the liquid fraction determined in the carrageenan-free extracts.

	Treatment Temperature (°C)				
Element (mg/L)	110	120	130	140	160
As	0.41 ^a^	0.45 ^a^	0.55 ^a^	0.45 ^a^	0.45 ^a^
Ca	6.85 ^c^	31.1 ^a^	10.1 ^b^	9.89 ^b^	9.68 ^b^
Fe	0.08 ^a^	0.08 ^a^	0.13 ^a^	0.07 ^a^	0.07 ^a^
I	1.09 ^d^	1.57 ^c^	2.43 ^b^	2.64 ^b^	4.02 ^a^
K	42.3 ^e^	95.1 ^a^	56.7 ^b^	50.9 ^c^	45.3 ^d^
Mg	33.0 ^d^	66.7 ^a^	46.1 ^b^	37.8 ^c^	37.9 ^c^
Na	362 ^d^	534 ^a^	505 ^b^	422 ^c^	432 ^c^
P	17.7 ^d^	22.1 ^a^	21.0 ^b^	19.0 ^c^	22.7 ^a^
Zn	0.70 ^b^	0.72 ^a,b^	0.75 ^a^	0.69 ^b^	0.70 ^b^

In all cases, standard deviations were < 3%. Data values in a row with different superscript letters are statically different (*p* ≤ 0.05).

**Table 3:** Protein and total carbon content of the residual solids.

Temperature	Proteins (%, d.b.)	Carbon (%, d.b.)
RS120	14.25 ± 1.94	27.18 ± 2.18
RS140	23.14 ± 0.26	37.11 ± 0.06
RS160	25.94 ± 0.45	40.27 ± 0.20
RS180	27.21 ± 0.41	40.89 ± 0.51
RS200	31.33 ± 0.37	43.32 ± 0.02
RS220	26.60 ± 0.39	45.39 ± 1.51

## Data Availability

Data are contained within the article or Supplementary Materials.

## Supplementary Materials

The following supporting information can be downloaded at: www.mdpi.com/article/10.3390/md21020083/s1, Figure S1: Influence of hydrothermal treatment temperature on the GPC profiles of *Sarcopeltis skottsbergii* crude extracts; Figure S2: Influence of hydrothermal treatment temperature on the GPC profiles of *Sarcopeltis skottsbergii*-isolated carrageenan; Figure S3: Influence of hydrothermal treatment temperature on the composition of crude carrageenan.
