# Supplementary material for: Microwave-Assisted Extraction of Carrageenan from Sarcopeltis skottsbergii
Source: Mar Drugs. 2023 Jan 25;21(2):83. doi: 10.3390/md21020083 (PMC9961692; doi:10.3390/md21020083)
Supplement: Supplementary file 1 [file marinedrugs-21-00083-s001.zip › marinedrugs-2115675-supplementary.pdf]

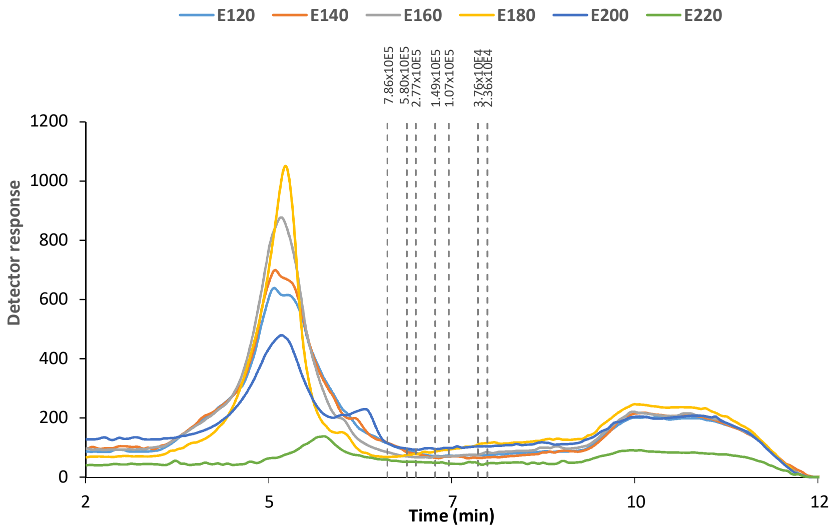

Figure S1. Influence of hydrothermal treatment temperature on the GPC profiles of *Sarcopeltis skottsbergii* crude extracts.

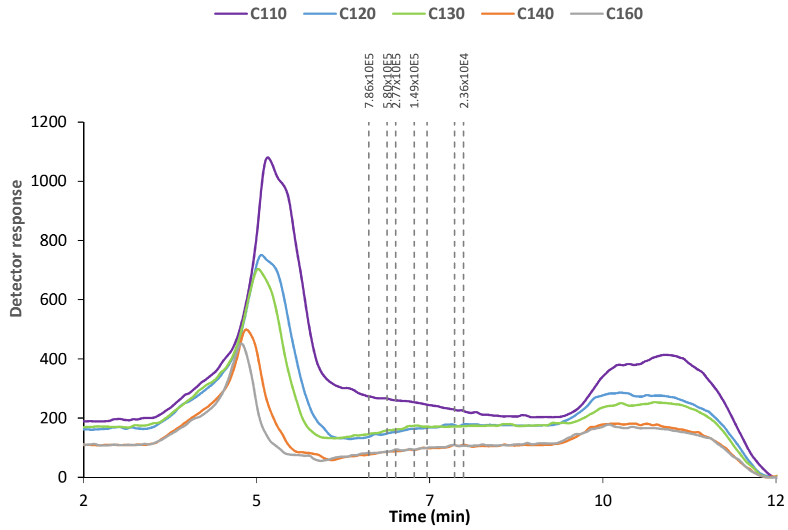

Figure S2. Influence of hydrothermal treatment temperature on the GPC profiles of *Sarcopeltis skottsbergii* isolated carrageenan.

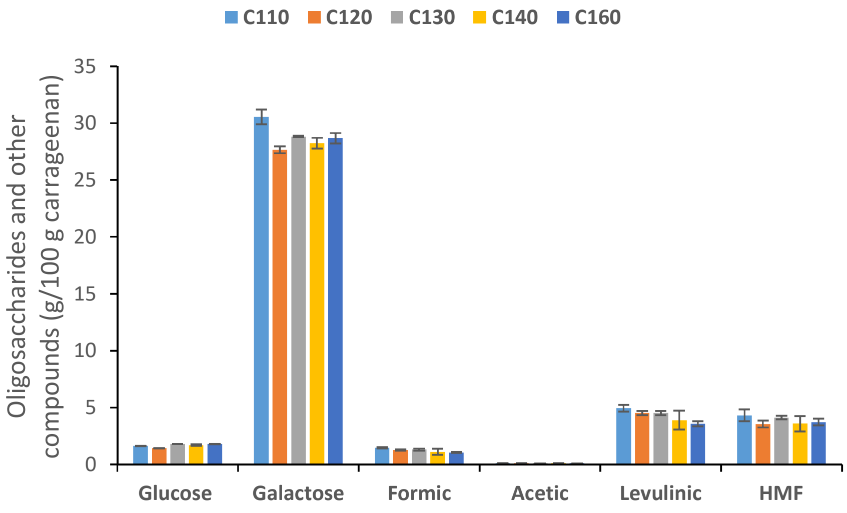

Figure S3. Influence of hydrothermal treatment temperature on the composition of crude carrageenan.
